# 125 When a Cancer Patient Gets Burned: Ethical Considerations Regarding Treatment, Privacy, and Distress

**DOI:** 10.1093/jbcr/iraf019.125

**Published:** 2025-04-01

**Authors:** Marcie Lambrix, Sharon Quallich, Casey Kohler, Monica Gerrek

**Affiliations:** MetroHealth System / Case Western Reserve University; MetroHealth; MetroHealth; MetroHealth System / Case Western Reserve University

## Abstract

**Introduction:**

Ethical issues in medicine are often analyzed using the principles of biomedical ethics: respect for autonomy, beneficence, non-maleficence and justice. However, these principles often conflict with one another, particularly when it comes to treatment decisions for medically and socially complicated patients, a common occurrence in burn units (Gerrek, 2018). In this presentation we consider such a case and discuss the ethical issues the burn team was challenged with as they navigated the clinical care of a single mother who also had an unforgiving cancer diagnosis.

**Methods:**

The patient was a 40-year-old female with 11% TBSA 2nd degree partial thickness burns along her lower extremities after slipping in hot grease while cooking. She also had a complex medical history of hereditary cancer with a poor prognosis. Despite this, she wanted to move forward with grafting and shared that she did not want her family to know about her cancer.

**Results:**

The patient had two adult children who did not live with her and eight younger children who did live with her. She reported being close with her father. The patient considered herself to be highly religious and had a very strong faith. Despite many discussions with various providers about the severity of her cancer and the grim prognosis, she believed her faith was helping her “get better”, despite significant pain and the amount of pain medication she was on. The burn, oncology, and palliative teams along with a hospital chaplain met with the patient to discuss her wishes regarding her medical situation. She remained steadfast in her choices not to discuss her cancer with any family and to move forward with grafting.

**Conclusions:**

The burn team contacted clinical ethics to discuss the patient’s situation and their distress surrounding it. Key ethical considerations that emerged included the obligation to offer grafting given her grim prognosis. Though the patient believed she was healing, the team worried that grafting may cause her even more pain and were concerned that the potential burdens outweighed the potential benefits of treatment. In addition, the team expressed discomfort that the patient was choosing not to discuss her cancer and its hereditariness with any family member. They worried that her children may share the genetic marker(s) and be at greater risk of developing cancer which they may never know about. The team wondered how they might navigate respecting patient autonomy and privacy as it conflicted with the ethical principles of non-maleficence and beneficence and what, if any, obligation did they have to the patient’s family.

**Applicability of Research to Practice:**

This case highlights the ethical complexity of navigating burn care for medically and socially complicated patients, provides a course for addressing these complexities, and underscores the need for teams to have resources for dealing with the distress in such situations.

**Funding for the Study:**

N/A

